# Combining Experimental Data and Computational Methods for the Non-Computer Specialist

**DOI:** 10.3390/molecules25204783

**Published:** 2020-10-18

**Authors:** Reinier Cárdenas, Javier Martínez-Seoane, Carlos Amero

**Affiliations:** Laboratorio de Bioquímica y Resonancia Magnética Nuclear, Centro de Investigaciones Químicas, Instituto de Investigación en Ciencias Básicas y Aplicadas, Universidad Autónoma del Estado de Morelos, Cuernavaca, Morelos 62209, Mexico; reinier.cardenazmen@uaem.edu.mx (R.C.); yadira.meunier@uaem.edu.mx (J.M.-S.)

**Keywords:** integrative structural biology, experimental techniques, computational methods

## Abstract

Experimental methods are indispensable for the study of the function of biological macromolecules, not just as static structures, but as dynamic systems that change conformation, bind partners, perform reactions, and respond to different stimulus. However, providing a detailed structural interpretation of the results is often a very challenging task. While experimental and computational methods are often considered as two different and separate approaches, the power and utility of combining both is undeniable. The integration of the experimental data with computational techniques can assist and enrich the interpretation, providing new detailed molecular understanding of the systems. Here, we briefly describe the basic principles of how experimental data can be combined with computational methods to obtain insights into the molecular mechanism and expand the interpretation through the generation of detailed models.

## 1. Introduction

One of the main aims in molecular biochemistry is to obtain mechanistic insights into the function of biomolecules. To accomplish this, researchers must design experiments that provide new information about the molecule in question by using a variety of biochemical and biophysical techniques. Subsequently, the experimental data have to be correlated with the specific characteristics of the molecule under study. This process is sometimes a straightforward interpretation, but in many others cases, it is difficult to decipher the molecular meaning of the data. Consequently, one of the main roles of an experimentalist is to interpret the data to obtain new information on a specific molecular mechanism based on the results.

With the advent of new computational methods, one of the experimentalist desires is to be able to incorporate the experimental data into a detailed representation of the different mechanisms using in-silico modeling to assist and enrich the interpretation. This conjunction could provide researchers with a new detailed molecular understanding and allow for the proposal of more complete mechanisms.

As a matter of fact, this has already been central to the development of biochemistry in the determination of structures by X-ray crystallography and nuclear magnetic resonance (NMR). In both these techniques, the experimental data (dispersion patterns and distance restrains) are combined throughout different computational protocols to propose a structure model that is compatible with the data [1,2]. However, it would be desirable to perform similar protocols with other experiential methods, not just to compute a static structure, but to integrate new data to understand new mechanisms, dynamics, and functions.

Recently, an integrative structural approach has been gaining increased attention, where the main idea is to integrate multiple experimental and computational methods to yield structural models of complex biomolecules (for some recent reviews, see [3,4]). This approach has already provided us with some remarkable results such as the structure and functional analysis of the nuclear pore complex [5] or the architecture of the 26S proteosome [6]. Although a static structural characterization of the different molecules provides structural snapshots reflecting functional activity, a better understanding of the mechanism of action requires experimental data measuring molecules undergoing structural transitions, binding, dissociation, and conformational fluctuations.

In this review, we briefly describe, from an experimentalist point of view, the basic principles and examples of how experimental data can be included into a computational model to obtain information about the molecular mechanism and functions beyond the static structure. We list some of the most widely used techniques, the measured biochemical variables, and some of the strategies to combine the experimental data with computational methods to expand the interpretation through the generation of detailed models. We do not intend to provide an exhaustive summary of all the methods, but rather to lay the foundations for future research. Although this review focused on proteins, many of the described approaches should be applicable to other molecules.

## 2. Basic Strategies to Integrate Experiments and Computational Methods

Although the combination of computational methods and experiments has been used for a long time, for instance, computational approaches have relied on experimental data to calibrate the force fields [7,8] while experiments have used computing power to process and analyze the data, this review focused on the use of computational methods to assist in the interpretation of experimental results.

This combination of methods can use four major different strategies:

(i) Independent approach. Experimental and computational protocols are performed independently, and then the results of both methods are compared. The first step in a molecular simulation would consist of sampling different conformations, which can be performed using a detailed atomic or coarse-grained representation (less detailed). The sampling protocols can be molecular dynamics (MD), Monte Carlo simulation (MC), or any other sampling technique (Table 1) (reviewed in [9,10,11]). In the best case scenario, computational models and experimental data correlate and complement each other. However, on some occasions, the biomolecular process under investigation is a “rare” event, and therefore, successfully sampling this event using a simulation technique requires a global search of the entire conformational space, which could be challenging. To solve this problem, several variations to enhance the sampling of conformations such as replica exchange molecular dynamics, metadynamics, and accelerated MD have been developed [10]. However, even with these advanced techniques, the sampling and accuracy of the generated structures are still bound by the limits of the force field and the theoretical model used, and sometimes the experimental data and the simulation do not correlate.

**Figure 1:**
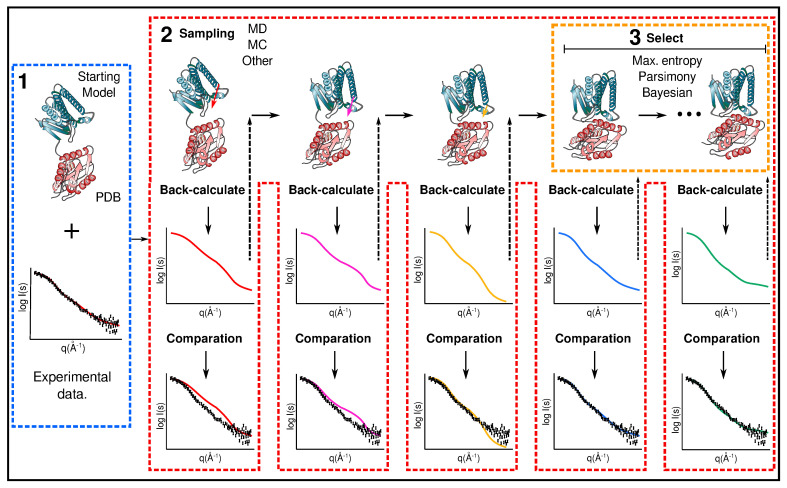
Schematic representation of the different steps in the guided simulation approach. (1) First, the experimental data are acquired and a structural model of the protein is selected. (2) Then, the experimental data are used as a restraint in the computational sampling protocol. This approach involves evaluating (back-calculated and comparing) each model during the simulation. As a result of this, the sampling space is reduced, and only conformations that correlate with the experimental data are sampled. (3) Finally, the conformers that best describe the data are selected.

**Figure 2:**
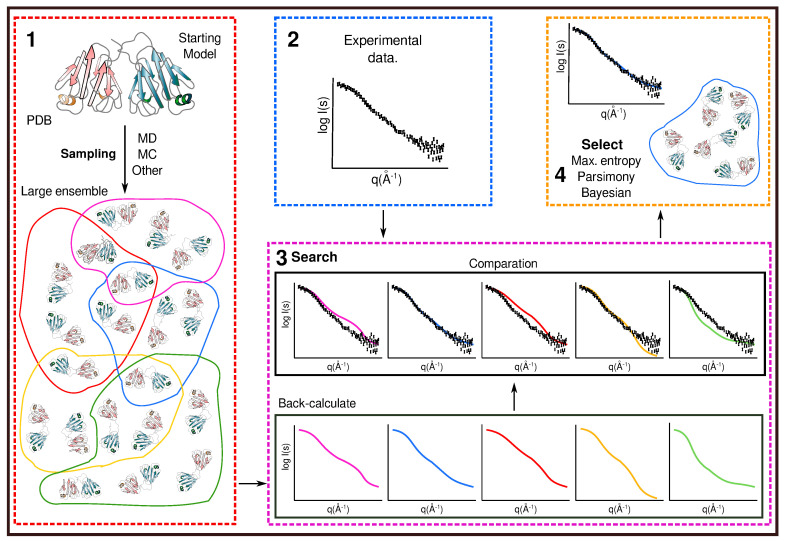
Schematic representation of the different steps in the search and select approach. (1) First, the computational sampling protocol is performed to generate a large pool of different conformation. (2) Independently, the experimental data are acquired. (3) In the search step, different ensembles of the molecule are used to back-calculate the biophysical variable to compare with the experiential data. (4) Finally, the ensemble that correlates better is selected based on a specific protocol to describe the experimental data.

This independent approach has been by far the most explored method, and although extremely powerful, we are more interested in a more integrated approach.

(ii) Guided simulation (restrained) approach. In a guided simulation, data obtained through experiments are used to effectively guide the three-dimensional conformation sampling in the computational method. This is usually done by the addition of external energy terms related to the experimental data into the computational protocol (restraints) (Figure 1). Each restraint has its target value (experimental distribution), against which the back-calculated values would be compared in each simulation step [10,13,14]. Since the guided methods involve evaluating the models during the simulation, they need to be implemented directly in the software. This type of guided simulation has been used in programs like CHARMM [15], GROMACS [16], Xplor-NIH [17] and Phaistos [18], among others.

(iii) Search and select (reweighting) approach. In a conceptually different strategy, the computation method is performed first to generate a large number of different conformation molecules (large ensemble), and then the experimental data are used to filter (search and select) the results (Figure 2). Only conformations that correlate with the experimental data are selected [13]. The generation of the initial pool of conformations can be performed by any of the simulation sampling techniques already mentioned. Sometimes, even less computational demanding protocols are used, such as generating a large pool of random conformations (MESMER [19] and Flexible-meccano [20]) or simulated annealing (Xplor-NIH [17]). Then, different protocols based on maximum entropy or maximum parsimony are used to select conformations that fit the data [12,13].

(iv) Guided docking. A different category of computational method would be molecular docking, which refers to methodologies that predict the final structure of a complex, starting with the structure of the two free molecules. Docking protocols are composed of two basic steps, a sampling algorithm to generate different binding conformations (poses) and a scoring process that assesses the quality of each pose (for some recent reviews, see [21,22]). In guided docking, the experimental data are used to help define the binding sites. In principle, the experimental data can be used either in the sampling or the scoring process [23]. Some docking programs that are able to incorporate the experimental data are HADDOCK [24], IDOCK [25] and pyDockSAXS [26].

It should be noted that many of the experimental biophysical techniques report average values over many molecules and long periods of time. Consequently, a better correlation has often been observed with back-calculated data from an ensemble of conformations than with data from just a single conformer. All of the strategies listed above can be used to obtain an ensemble of conformations that are compatible with the set of experimental average values [12,13,27]. Hence, a large number of programs have been created to select ensembles that fit the experimental data. For instance, ENSEMBLE [28], X-EISD [29], BME [30], and MESMER [19] were used to select conformations that matched data from several different experiments. These approaches differ in the way in which the initial ensemble is generated as well as in the algorithm used to search and select the final ensemble.

The use of one strategy over the others would depend on the specific characteristics of each study. However, we can list some of the advantages and disadvantages that would make it more likely to choose one approach over the other. The computational sampling in the independent approach is not restricted to sample a specific region of the conformational space and therefore can provide information on “unexpected” conformations. Additionally, if one is interested in the specific sequential pathways of a process, un-bias sampling can provide a plausible pathway based on the physical model in which the computational method is based. On the other hand, one of the main advantages of the guided simulation approach is that the restraints considerably limit the conformational space and, in principle, the “observed experimental” conformations are sampled more efficiently. The main disadvantage of this approach is that the experimental data have to be implemented as a restraint during the sampling, and this could be a difficult task and in most cases would require certain computational knowledge.

In the search and select approach, the sampling process is uncoupled and is performed independently of the experiential data, and consequently the integration of different methodologies and more than one experimental restraint is simpler. Furthermore, it is possible to incorporate new experimental data without the need to generate a new confrontational ensemble. One of the drawbacks would be that the initial pool must contain the “correct” conformations, and therefore it also requires a large sampling of the conformational space, however, several programs that easily generate a large pool of structure have been developed. Finally, if what one wants is to understand the formation of a complex, the best approach would probably be the use of guided docking.

Another potential challenge is deciding which computer program to use, and even though different software would be more useful in different situations, with the non-computation specialist in mind, we undertook the task of testing some of the different software available. We did not attempt to present an exhaustive list of all the existing programs and their features. In Table 2, we enlist some of the programs that are able to integrate experimental data, are freely available, and are moderately easy to install and use. For a larger table with brief description of software see Table S1, Supplementary Materials.

In order to be able to integrate the experimental results into these approaches, it is necessary to compare the experimental data with a back-calculated value from the computational method. Therefore, it is necessary to be able to interpret the experimental results as a biophysical variable like distance, volume, or any other structural parameter, and at the same time, be able to compute the same variable (distance, volume, or any other structural parameter) from a three-dimensional model (PDB file). In the next section, we briefly describe some of the experimental methods that have been combined with computational models to obtain new molecular insights.

## 3. Nuclear Magnetic Resonance

**Figure 3:**
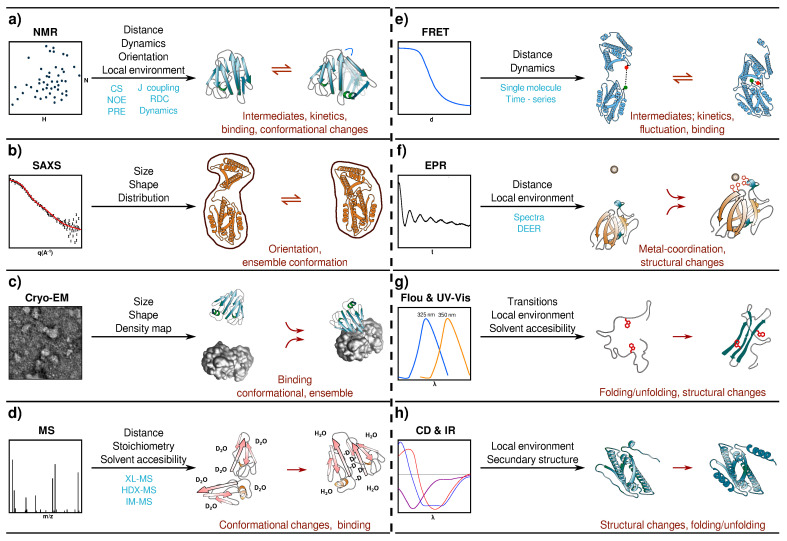
Schematic representation of the different experimental techniques that are usually combined with computational methods. The biophysical observable and some of the new information processes are listed. (**a**) Nuclear magnetic resonance (NMR). (**b**) Small angle X-ray scattering (SAXS), (**c**) Cryo electron microscopy (Cryo-EM) (**d**) Mass spectrometry (MS), (**e**) Förster resonance energy transfer (FRET), (**f**) Electron paramagnetic resonance (EPR), (**g**) Fluorescence and UV–Vis (Fluo & UV-Vis), (**h**) Circular dichroisms and infrared absorption (CD & IR).

Nuclear magnetic resonance (NMR) spectroscopy is probably the one experiential technique that has been most often and successfully integrated with computational methods [31,32,33,34,35]. This is probably due to the site-specific information, which allows each nucleus to be monitored and a simple correspondence with the atomic information obtained from in-silico models (Figure 3a). From all the variables that can be measured with NMR, the most commonly and simply obtained experimentally in biomolecular study is the chemical shift (CS) (the resonance frequency), which is determined by the nucleus’s local electronic environment. This chemical environment is exquisitely sensitive to change, and in general terms, it depends on the structural conformation, dynamics, or interactions [36]. Due to this, the chemical shifts, in principle, could provide a form to monitor a large range of different information from molecular processes.

There are several programs that predict CS based on a three-dimensional structure using different methods (for instance ProCS15 use DFT quantum mechanics [37]; SHIFTX2 [38], and SPARTA+ [39] use empirical approximations). And while there are usually higher discrepancies with the back-calculated H and N, most of them perform very well when computing the carbons chemical shifts. These developments have enabled the integration of CS with computational methods in which structures obtained in-silico are used to predict the CS and then compared with the experimental data. CS have been implemented as restraints in different types of simulations including MD [16], MC [40], simulated annealing [17]), or integrated in search and select programs (CS-Rosetta [41], ENSEMBLE [28], and MESMER [19]). In addition, CS data have proven very useful to refine structures or to make new structural models [42].

In an original protocol proposed to describe partially folded states, changes in CS were used to describe intermediates states [43]. In this approach, the CS that did not change between the native spectrum and a partially unfolded state spectrum were used to implement “fictional native-like” restraints, whereas residues with different CS were let to vary freely under a force field in an attempt to obtain information on intermediate conformations. The protocol was used to model the partially unfolded state of the photoactive yellow protein.

Another NMR observable that has been frequently combined into computational methods is the nuclear Overhauser effect (NOE). The NOEs provides direct evidence of through-space transfer of magnetization from a nearby nucleus, and therefore the intensity of the obtained signal is proportional to the distance between two nuclei [2]. The implementation of NOEs as restraints in computational methods is therefore straightforward; nevertheless, obtaining and interpreting NOEs in experiments is usually time consuming. Another drawback is that NOE data provide only short-range distances and are usually only observed for the most stable population. Additional distance restraints can be obtained via the paramagnetic relaxation enhancement (PRE) effect. To measure PRE, it is necessary to have a paramagnetic label, and then the relaxation depends on the inter-nuclear distance between the nucleus and the ion. PRE has emerged as an important development to integrate with computational methods (see review [44]).

The NMR couplings (*J* and dipolar) provide angular and relative orientations of bond vectors. The J coupling has been used as a restraint or as control for many computational methods [8,45,46], while residual dipolar coupling (RDC) have been extensively used over the last two decades, most likely due to the easiness to measure them and the fact that great correlation can be achieved with back-calculated RDC from structures [47]. RDCs have been integrated with multiple computational protocols, but one of the most useful has been to select structural ensembles that correlate with experimental data, mostly for intrinsically disordered proteins (IDP) [48]. In this approach, RDC data obtained from IDPs are compared with RDC back-calculation from a large randomly generated ensemble, and then the conformational ensemble that matches the experimental data is selected.

A particularly relevant example of the use of RDCs for IDPs involves studies of the C-terminal domain of the Sendai virus nucleoprotein [49]. In this study, the analysis of the experimental data through a “search and select” protocol resulted in an ensemble that contained completely unfolded states, but also conformers with a residual secondary structure, proving that the protein exists as a dynamic conformational ensemble of states, among which they can interconvert. Studies of this system were taken a step further by studying the interconversion kinetics between the different conformers by MD CS guided simulation [50].

Finally, dynamic data have also been complemented with computational methods. This is interesting because it provides a direct correlation with some of the variables obtained directly from molecular simulations. Different NMR experiments that provide dynamic information in different time scales are used to characterize the molecular motion at atomic resolution [35]. The most common are related to some relaxation rates of measurement, in which a series of experiments with different parameters are collected and then each signal is fitted to some equation. Most of the integration with NMR dynamics has consisted of comparing the results from both techniques such that the in-silico approach helps to interpret the experimental data (among recent examples [51,52]).

## 4. Small Angle X-Ray Scattering

Small angle X-ray scattering (SAXS) has emerged as an important method to incorporate experimental data into computational models. SAXS provides information on the biomolecular shape, which can then be used to gain insight into biomolecular pathway interactions, assembly states, ensemble conformational populations, and dynamics of disordered systems, among others (Figure 3b) [53,54].

Although SAXS is a low-resolution technique, due to the fact that it is performed in solution and does not have a size limitation, it is a perfect complementary technique for NMR and X-ray crystallography. One extremely valuable form of information that can be obtained by SAXS, that is not easily determined using other techniques, is the overall orientation. The obtained structural envelopment allows us to estimate a reasonable orientation of domain, sub-units, or even alternative conformations sampled by a molecule in solution [53]. Additionally, recent advances in time-resolved SAXS experiments provide an opportunity to obtain information of fast processes [54].

In a SAXS experiment, the average intensities of X-rays scattered by a sample are measured at small angles and expressed as a function of the scattering vector amplitude. The SAXS profile are traditionally analyzed by separating the data in distinct regions to obtain biophysical variables like the radius of gyration, maximum particle size, volume, and mass [54]. Even though integration with computational methods can be done using any of these biophysical variables, most of the integration is done using the complete scatter profile.

Theoretical SAXS profiles can be back calculated from the coordinates of atomic models and then compared with the experimental SAXS curves [54,55]. It is important to note that since the data are spherically averaged, different models may have similar SAXS profiles, all of which could be consistent with the experimental data. There are several programs to back-calculate theoretical scattering profiles from a PDB structure like Fast-SAXS [56], CRYSOL [57], and FoXS [58].

This synergy between computational methods and the analysis of the data provides the basics to implement these techniques as a variable into a computational method. One possible approach is to use the profile to directly guide the simulation. In this case, sampling conformations are generated using MD, MC, or other computational techniques, and then, the deviation between the observed and predicted SAXS profiles are evaluated for each step, effectively guiding the simulation [59]. This has been implemented using Bayesian and maximum entropy approaches with full MD atomic simulations, coarse grained simulations, elastic network, replication modeling, and metadynamics [54,60,61]. In an interesting protocol proposed to find intermediate pathways, two different states measured by SAXS (initial and final states) were used to guide the simulation from the initial state toward the final state, trying to find transitional conformation [62].

Alternatively, the experimental SAXS profile can be used to filter previously generated conformations to obtain a representative ensemble average. Here, the theoretical scattering profile of each conformer is computed, and a selecting step followed to obtain the best fitting description of the data. Several methods have been proposed to select the ensemble that best fits the experimental data [63] even for IDP [64,65]. Among the most frequently used methods are basis-set supported SAXS (BSS-SAXS) [66], the ensemble optimization method (EOM) [60,67], and the minimal ensemble search (MES) [68].

In a similar way, small angle neutron scattering (SANS) can be used to correlate the experimental data within silico methods. Even though SAXS and SANS share similar principles, neutrons have different scatter properties and therefore can provide complementary information. For instance, it has been shown that the combined use of SAXS and SANS can help in the interpretation of the data [69,70].

## 5. Cryo Electron Microscopy

Over the past decade, advances in cryo electron microscopy (Cryo-EM) and image processing have expanded the range of targets, becoming one of the most important methods to characterize molecular structures. Cryo-EM has gone from being useful mainly to determine the relative orientation of known structures, to achieving near-atomic resolution (Figure 3c) [71,72].

This advance has allowed the structural characterization of complex biological systems, and in contrast to X-ray and NMR, it requires small amounts of the sample, sample crystallization is not necessary, it provides long-distance information, and it is applicable to large molecular weight systems [73]. This technique is now used to uncover how proteins assemble or even to find drug targets by docking [74]. In addition, it is applicable to cell membranes [75], which have been very elusive for other techniques. One interesting feature of Cryo-EM is that it is usually performed in a thin layer of fast-frozen solution, and therefore, the particles’ orientation is random. This procedure could provide a snapshot of alternative structural conformations accessible for the molecule [76].

A number of approaches have been proposed to model structures based on Cryo-EM density maps that go from rigid body fitting of known structures to flexible fitting and de novo protein structure modeling [77,78,79]. Most programs generate models that minimize the deviation between the observed density map and one predicted from the structure; this can be done using MD, MC, or normal mode methods, among others. One of the most widely used is molecular dynamics flexible fitting (MDFF), which has been implemented in IMP or Rosetta [80,81]. Several recent papers have presented a comprehensive overview of how computational methods are used to assist structure refinement (see review [77,82]).

Most of the integration of Cryo-EM with computational methods has been done to refine a static structure into density maps. Nevertheless, in a nice example of using experimental data with computational methods to study dynamic changes, Cryo-EM structures of immature dengue virus bound to a human monoclonal antibody and MD simulations were used to show the mechanism by which the antibody facilitates the dissociation of pr proteins present in the particles [83]. These data provide evidence that binding of the antibody to the pr protein induces dissociation of the pr protein from protein E at low pH. This exposes the E protein fusion loop, enhancing virus interaction with endosomes, allowing the immature particles to be infectious.

## 6. Mass Spectrometry

Mass spectrometry (MS) is a very powerful technique with many applications. Nevertheless, until recently, it has not been commonly combined with computational methods (Figure 3d) [84]. This integration has mainly taken place in three different ways: cross-linking coupled to mass spectrometry (XL-MS) [85], which is a fast and efficient way to obtain distance restraints between pairs of residues; hydrogen/deuterium exchange (HDX-MS) [86], where the region of solvent accessible residues are determined; and native ion mobility (IM-MS) to study the assembly and disassembly pathway of whole complexes [87]. The main advantage of this technique over others is that it is potentially applicable to any protein system, regardless of size or flexibility including membrane assemblies.

XL-MS consists of incubating a system with a bi-functional cross-linker agent followed by proteolytic cleavages and then MS analysis. In this manner, pairs of residues that are cross-linked are identified, and therefore residues that are close together in space are marked [85,88]. An estimation of the distance can be proposed based on experimental conditions and then, the data can be incorporated into the structural simulations as distance constraints between carbons of the corresponding residues. Some of the advances have been reviewed in [88,89].

On the other hand, HDX-MS consists of exposing the sample to a deuterated solvent followed by proteolytic cleavages and MS analysis. Labile hydrogen atoms exchange with deuterium. The rate of this process is influenced by the chemical features of the exchanging groups, but also by the structure conformation. For each peptide identified after proteolysis, deuterium incorporation is then determined as the change in peptide mass over time, and a protection factor (P) is calculated based on the difference between the measured and the expected exchange for an unfolded protein [90]. Several semi-empirical models have been proposed to define a relationship between the protein conformation and the corresponding hydrogen exchange. One of the phenomenological approximation models that have shown a good correlation defines the protection factor as a linear combination of the H bonds and the packing, defined as the number of heavy atoms within 6.5 Å of distance from the amide hydrogen (Equation (1)) [91].

lnP = β_1_ H_bonds_ + β_2_ C_paking_ (1)

where β_1,2_ were experimental adjusted parameters (β_1_ = 2 and β_2_ = 0.35).

With the implementation of these models, HDX-MS data can then be predicted from a structure and therefore HDX-MS experiments can be used to guide simulation, docking approaches, or search and select protocols [86,92,93].

For instance, HDX-MS studies of the viral helicase P4 a 6-subunit ring with MD simulation, identified a rapid equilibrium between different conformations [94]. The experimental exchange was significantly faster than the predicted exchange for the hexamer based on MD simulation, and it was only possible to find good correspondence with the experimental data if the MD predicted exchange for the monomer was included in the interpretation. Furthermore, the analysis also showed that other parts of the proteins were undergoing conformational changes.

More recently, IM-MS has been used to study composition, stoichiometry contacts. and interaction in molecular ensembles [87,95]. Computational simulation has been used to understand the extent and under what conditions the condensed-phase properties are preserved upon transfer into the gas phase. Novel research is emerging using MD simulations to provide insights into the behavior of molecules in the experiments. For some reviews, see [93,96]. 

## 7. Förster Resonance Energy Transfer

Förster resonance energy transfer (FRET) is a widespread spectroscopic technique to measure specific conformational changes in macro-molecular systems. FRET has provided insights into the folding of proteins, assembly and disassembly of complexes, enzymatic cycles, structure changes, binding and dynamic processes (Figure 3e) [97,98]. Contrary to other techniques (NMR, X-ray crystallography, CryoEM), FRET is quite simple to use, is fast, and has time resolve spectroscopy capable of covering wide timescales. Furthermore, FRET can be performed as a single molecule experiment, which makes it possible to distinguish static states (multiple static conformation) and dynamic heterogeneities (inter-converting states) and provide kinetic information (reviewed in [99,100]).

The direct energy transfer from a donor (D) to an acceptor (A) fluorophore is inversely proportional to the sixth power of the distance between them. The measurements can only be performed for inter-dye distances, and therefore this requires labeling the system [100]. This distance measure can be easily integrated as a distance restraint in any computational techniques such as MD, MC, and docking, making FRET a simple technique to detect conformational changes in a system [101,102].

In an impressive example of the functional insights from FRET integration with computer methods, different conformational states of the T4 lysozyme were investigated [103]. Using a hybrid FRET approach composed of multi-parameter detection, correlation spectroscopy, time resolved experiments, and computer simulation, three conformational states in fast kinetic exchange were characterized. These data support the existence of a conformational state never seen before.

Most of the single-molecule measurement data have been combined with computational methods as a distance distribution, leaving the temporal dependency mostly unused. However, recently, new methods have been proposed such as the so called “time-series of single-molecule” based on machine-learning [104,105]. In this method, two MD simulations are performed, in the first, the transition between states is clustered, and in the second, the integration of the experimental time-series data re-calibrates the parameters, which then permits simulating the time-series accurately.

## 8. Electron Paramagnetic Resonance

Electron paramagnetic resonance (EPR) spectroscopy is used to gain the information of paramagnetic molecules. It provides information on metal-proteins, free radicals, and protein function (Figure 3f). Nevertheless, most of the proteins do not contain paramagnetic ions (unpaired electrons), and therefore it is required to attach labels into specific parts of the molecules many times [106].

Computational methods have been widely used in conjunction with EPR to determine metal coordination in proteins, usually making use of density functional theory. These calculations are often difficult and provide mostly local information. More recently, several methods to simulate the complete EPR spectra using the MD trajectory have been developed [107,108]. Nevertheless, a breakthrough into the conformational computational techniques to understand fluctuation has been achieved using double electron–electron resonance (DEER) spectroscopy (also known as pulsed electron-electron double resonance) [109].

DEER uses dipolar interaction between pairs of un-pair electron spins to measure distances between the paramagnetic labels. The observable measure is a time-domain signal that is then converted into a distance distribution. To incorporate the restraint into a computational method, it is usually necessary to incorporate the distribution, which is related to the experimental uncertainty [110]. For DEER, there is not a clear consensus of the type of distribution that should be used [110]. Nevertheless, DEER data have been implemented in several protocols such as ensemble-biased metadynamics (EBMetaD) [111,112].

An alternative protocol, named restrained-average dynamics (RAD), has been proposed in which a MD simulation is directly guided with the DEER signal without transforming this to distance, thus avoiding the distribution problem [113]. This methodology was used to study the structural dynamics of the two domain VcSiaP proteins, in which a MD simulated ensemble was obtained. Interestingly, the ensemble did not contain a closed conformation, which allowed the authors to propose that this conformation is not accessible in the absence of substrate [113].

## 9. Fluorescence, UV–Vis and Infrared Spectroscopies

Fluorescence, UV–Vis, and infrared absorption (IR) techniques are some of the most popular spectroscopic methods to study function, structural transitions, folding, and dynamics in biomolecules. However, the many factors that affect the measurement, signal overlapping, and in general, the complexity of the process that gives rise to the signal, makes it difficult to properly interpret the spectra in terms of structural features and transition fluctuations.

Understanding of the process at the atomic level requires the use of complex quantum mechanics calculations, which are in general very computationally demanding. Nevertheless, over the years, different theoretical-computational methods have been proposed to predict the spectra; some have used semi-empirical relation [114], classical methods [115], or mixed quantum mechanics strategies [116,117] to provide information on the complex absorption–structure relation.

For instance, diverse phenomenological models have been proposed to predict the fluorescence emission wavelengths of tryptophans [114,118] or even the decay rates [119]. Some of the models that correlate well are based on electrostatic interaction of the indole group or on the solvent-accessible surface area [114]. This type of model allows for an easy interpretation of the fluorescence spectra of proteins using computational methods.

Even though the UV–Vis absorption spectra are much less sensitive to the local environment than fluorescence, some studies have tried to reproduce the spectra using computational approaches (Figure 3g). For instance, a hybrid approach of molecular dynamics and ab initio techniques was used to calculate the absorption spectra of tryptophan in Barnase [120]. In a very interesting study, the use of computational calculation allowed them to explain the experimental observation where a monomer protein lacking aromatic amino acids presented an absorbance between 250–400 nm. Calculating the corresponding transitions from MD trajectories using time dependent density functional theory, it was shown that the charged sidechain amino-carboxylate groups in the Lys-Glu residue was responsible for the absorbance [121].

IR absorption spectra has been mostly used to determine the secondary structure of peptides and proteins (Figure 3h) [122]. Nevertheless, some computational approaches have shown that the shift in frequency is strongly dependent on the number of hydrogen bonds to the amide oxygen atom or the amide NH group [117]. Recent approaches to predict the IR spectra have used mixed QM methods to describe, at the atomistic level, the vibrational behavior. In a nice study, the experimental and the calculated time-resolved IR spectra at multiple frequencies for the fast-folding of GTT35 protein was compared. The analysis shows that the IR signal is consistent with folding through intermediates and allows the determination of the corresponding kinetic parameters [123].

The ability of the reproduction of the experimental spectra using computational methods provides an atomic detail description that was not accessible by only the experimental data.

## 10. Other Techniques

The implementation of computational methods with other techniques has been reduced, mostly due to the difficulty of defining a clear biophysical variable from the experiment or being able to back-calculate this variable from computer coordinates. Nevertheless, the implementation would be performed in a similar manner; making use of a guided simulation using the “search and select” approach or a docking protocol.

For instance, the development of software that predicted circular dichroisms (CD) spectra from the structure (DichroCalc [124] or PDB2CD [125]) opened the door to implement this technique into the simulation of selected ensembles (Figure 3h). Even though the correlation is not great yet, it can be used to estimate folding pathways using, for instance, CD coupled to stop flow methods [126].

Another interesting experimental technique to combine with computational methods is high-speed atomic force microscopy (HS-AFM). HS-AFM directly observes biomolecular topological and dynamics at near the physiological condition and at the single molecule level [127,128]. Recently, HS-AFM was used to study the unbinding of streptavidin–biotin complex and compared to MD simulations [129]. Additionally, it was used in conjunction with coarse-grained MD simulations to fit the AFM image, and this proposed methodology allows us to infer from the MD the ionic concentration of the solution and the timescale of the different conformations [130].

Perhaps the easiest way to start combining different experimental techniques with computational descriptions would be the use of ensemble select and search protocols to choose the best structures that describe the experimental measured variable. An interesting software that already allows us to implement different measures in the selection of the best representing ensemble is MESMER [19]. It is possible to include new measure variables as a table and then, by using external software to back-calculate the same variable from the generated ensemble, the software allows us to compare the values and then choose the best fit.

## 11. Conclusions

Since the conformation of biomolecules undergoes variations with time and functional state, providing a detailed molecular description that incorporates these changes based solely on experimental results is a difficult task.

The integration of experimental data with computational techniques allows us to obtain a detailed interpretation of the results that would not be achievable using only experimental methods.

We are certain that the integration and applicability of some experimental techniques with computational methods are going to continue, and we anticipate new developments and integration with other experimental techniques.

## Figures and Tables

**Table 1:** Glossary of some computational terms.

Computational Term	Brief Description
Molecular dynamics simulation	Sampling method. New conformations are generated by using Newton’s equations (Force field) [9].
Monte Carlo simulation	Sampling method. New conformations are generated by random perturbations, then the conformation is accepted or rejected based on some fixed criteria [9].
Docking methods	Computational method to predict complex formation. It consists of two steps the simulation, where different binding poses are sampled and the scoring, where the best binding pose is selected based on predefined rules [9].
Selection based on maximum entropy	This method selects the larger number of conformer (maximum entropy) that match experimental data [12].
Selection based on maximum parsimony	This method selects the minimum number of conformers (maximum parsimony) that can explain the experimental data [12].
Selection based on Bayesian	This methods combines the use of prior information and new evidence in the selection process [12].

**Table 2:** List of some of the available software used in the integration of experiments with computational methods.

Program	Accepted Experimental Data	Functionality	Availability	Ref
CHARMM	Distance */Cryo-EM	Molecular Dynamics simulations software.	www.charmm.org	[15]
GROMACS	NMR/Distance *	Molecular Dynamics simulations software.	www.gromacs.org	[16]
Xplor-NIH	NMR/SAXS/Cryo-EM Distance *	Structure determination software.	nmr.cit.nih.gov/xplor-nih	[17]
Phaistos	NMR/SAXS	Monte Carlo simulations software.	sourceforge.net/projects/phaistos/	[18]
Flexible-meccano	NMR /SAXS	Generate randomly conformers ensembles	www.ibs.fr/research/scientific-output/software	[20]
HADDOCK	XL-MS/HDX-MS/Cryo-EM NMR	Information-driven flexible docking approach	bianca.science.uu.nl/haddock2.4/	[24]
iDOCK	Distances *	Docking. Included on IMP	integrativemodeling.org	[25]
pyDockSAXS	SAXS	Docking with SAXS profile	life.bsc.es/pid/pydocksaxs	[26]
ENSEMBLE	SAXS/NMR	Ensemble selection software.	abragam.med.utoronto.ca/~JFKlab/#	[28]
X-EISD	NMR/SAXS/FRET	Ensemble selection software.	github.com/THGLab/X-EISD	[29]
BME	Distances */SAXS/NMR	Entropy ensemble selection software.	github.com/KULL-Centre/BME	[30]
MESMER	DEER/SAXS/ NMR/Other	Minimal ensemble Solutions to Multiple Experimental Restraints	github.com/steelsnowflake/mesmer	[19]

* Distance data can be from NOE, FRET, XL-MS, DEER.

## Supplementary Materials

The following are available online, Table S1: Additional tested software.
