# Supplementary material for: Combining Experimental Data and Computational Methods for the Non-Computer Specialist
Source: Molecules. 2020 Oct 18;25(20):4783. doi: 10.3390/molecules25204783 (PMC7594097; doi:10.3390/molecules25204783)
Supplement: Supplementary file 1 [file molecules-25-04783-s001.pdf]

We have tested some of the software to integrate experimental data with computational methods, from sampling to back-calculate biophysical values. From the large group of software available, we enlist in the following table some of the programs that can integrate experimental data, are freely available, and are moderately easy to install and use. We are not trying to present an exhaustive list of all existing programs and their features; rather, the aim is to serve as a starting point to help new users. For informational purposes only, we list the minimum data required to run each of the listed programs, as well as some of their specifics. However, in some cases, the program may require other inputs in order to implement more complex calculations.

**Software**	**Input data**	**Brief description**	**Difficulty to install and use[1]**	**Installation comments**	**Requirements**
		**Molecular Dynamics**			
GROMACS<br><br>www.gromacs.org	Distances<br><br>Angles	Molecular dynamic simulations software. The simulation can be performed with or without restrictions.<br>Possible restriction include: position, angle, dihedral, distance, and orientation restraints.<br>Available under the GNU General Public License.	+++	Download and installation from Ubuntu repository or download from the website.	- PDB.<br><br>- Instruction file.<br><br>- Restriction files.
CHARMM<br><br>www.charmm.org	DEER<br><br>Distances<br><br>RDC	Molecular dynamic simulations software, with a full set of energy functions, a variety of enhanced improved sampling methods and other advance techniques. CHARMM-GUI server can be used to prepare the inputs.<br>Reduced version for free.	++++	Registration necessary.<br><br>Command line program.	- PDB.<br><br>- Instruction file.<br><br>- Restriction files.
BILBOMD<br><br>sibyls.als.lbl.gov/bilbomd	SAXS	Conformational sampling using molecular dynamics to adjust the three-dimensional structure of protein domains by SAXS data.<br>Sampling is followed by ensemble selection using FoXS and the minimum ensemble selection protocol. Simulations are submitted from website.	+	Run from server.<br>Does not required installation.	- PDB.<br><br>- SAXS data.
Xplor-NIH<br><br>nmr.cit.nih.gov/xplor-nih/	NMR<br><br>SAXS<br><br>Distances	Structure determination program that includes tools to implement a wide variety of experimental data. Includes modules to perform monte carlo, molecular dynamics and minimization using torsion angles and cartesian coordinates.<br>Parameter files can be calculated from the amino acid sequence or the PDB file.	+++	Registration necessary.<br><br>Command line program.	- PDB<br><br>- Experimental data.<br><br>- Instruction file.

Software	Input data	Brief description	Difficulty to install and use[1]	Installation comments	Requirements
**Monte Carlo**					
Phaistos<br><br>www.phaistos.org	CS<br><br>SAXS<br><br>Distances	Monte Carlos framework using Markov chain approaches for simulations of proteins. It can incorporate different energy functions such as OPLS-AA, Profasi, and probabilistic Bayesian inference. Can use ProCS, ProCS15 and CamShift to predict the CS in a guided simulation.	++++	Requires to be compiled.<br><br>Need out-dates libraries.	- Amino acid sequence or PDB.<br><br>- Restriction files.
PROFASI<br><br>cbbp.thep.lu.se/ activities/profasi/	Distances<br><br>Secondary structure	Monte Carlo simulations proteins package originally developed for for folding and aggregation simulations. Simulations can be performed with arbitrary measurements as restrains such as: distance, torsional angle, radius of gyration or secondary structure.	+++	Requires registration on the site.	-Amino acid sequence or PDB.<br><br>- Restriction files.
**Guided Docking**					
HADDOCK<br><br>haddock.science.uu .nl	NMR<br><br>Other	HADDOCK consists of a collection of python and CNS scripts. It is mainly used for flexible docking incorporating experimental data. The coupling protocol consists of three successive steps: (i) minimization of rigid body energy, (ii) semi-flexible refinement in torsion angle space, and (iii) final refinement in explicit solvent refinement. Interface regions can be identified by mutagenesis, H/D exchange and cross-linkers or NMR.	++	Registration necessary.<br><br>Run from server.<br>Does not required installation.	-PDBs.<br><br>-Restriction files.
iDOCK<br><br>integrativemodeling. org	Distances	iDOCK allows docking proteins with experimental data very easily. The data included the residues and the minimal and maximal distance between the CA of each residue.	+++	Included on IMP, the Integrative Modeling Platform.	-PDBs.<br><br>-Restriction files.
PyDock-SAXS<br><br>life.bsc.es/pid/ pydocksaxs	SAXS	Web server for rigid-body protein-protein docking based on experimental data. It generates a 3D representation of a complex in solution by using two scoring functions. Evaluates the interaction surface of multiple docking poses, and assesses the capacity of each pose to describe the experimental curve of SAXS with the help pf CRYSOL.	+	Run from server.<br>Does not required installation.	-PDBs.<br><br>-SAXS curve of the complex.

Software	Input data	Brief description	Difficulty to install and use[1]	Installation comments	Requirements
**Ensemble modeling**					
ENSEMBLE<br><br>abragam.med.utoronto.ca/~JFKlab/	NMR<br><br>SAXS	Program to select an the weighted ensemble that best represents the unfolded state of a protein from a iterative fitting of the experimental data.<br>It can select base on CS, NOE, PRE, RDC, 3J couplings, hydrodynamics radius, protection factor and SAXS.	+++	Requires to be compiled.<br><br>Requires external software to generate Initial pool of structure, and back-calculate variables.	-Initial pool of structures.<br><br>-Parameter file.<br><br>-Experimental Data.
X-EISD<br><br>github.com/THGLab/X-EISD	NMR<br><br>SAXS	Python script to perform experimental inferential structure determination of ensembles for IDPs, using a Bayesian approach.<br><br>To run it, you must create a python file containing the instructions and file locations.	++	Download and decompress.<br><br>Initial pool of structures. Requires external software to generate Initial pool of structure, and back-calculate variables.	- Initial pool of structures.<br><br>-Experimental data<br><br>-Back-calculated data
BME<br><br>github.com/KULL-Centre/BME	NMR<br><br>SAXS<br><br>Distances	Python script that uses the Bayesian maximum-entropy approach to fit a simulation to experimental data.<br><br>The back-calculate data has to be calculated from the initial pool of structures.	++	Download and decompress.<br><br>Requires external software to generate Initial pool of structure, and back-calculate variables.	-Experimental data.<br><br>-Back-calculated data from simulation trajectory.
MESMER<br><br>github.com/steelsnowflake/mesmer	SAXS<br>NMR<br>Distances<br>Other	MESMER can be used to generates different protein conformations by randomization of backbone Phi/Psi angles, and then allows selecting a set of structures that fit the experimental data from the created ensemble. It is possible to incorporate almost any type of data. Graphical user interface – GUI	+++	Requires external software to back-calculate variables.	-PDB<br><br>-Experimental data

Software	Input data	Brief description	Difficulty to install and use[1]	Installation comments	Requirements
**Other**					
IMP<br><br>integrativemodeling.org/	SAXS<br><br>Cryo-EM	Platform that containing different modules that can be used to adjust simulations to experimental data: FoXS and MultiFoXS for SAXS data, as well as MiltiFit and CNMultiFit for cryo-EM data. IMP contains a large number of modules.	++	IMP can be installed on Linux, MAC, and Windows.	-Module specific.
CRYSOL<br><br>www.embl-hamburg.de/biosaxs/crysol.html	SAXS	CRYSOL evaluates the atomic structure of a macromolecule and fits it to experimental SAXS dispersion curves.	++	CRYSOL is included in a software called ATSAS.	-PDB.<br><br>-Experimental SAXS data.
MDFF<br><br>www.ks.uiuc.edu/Research/mdff/	Cryo-EM	MDFF is used to fit atomic structures in density maps. The method consists of adding external forces proportional to the gradient of the density map into a MD simulation of the atomic structure.	+++	Can be installed as VMD plugin or implemented on IMP.	-PDB.<br><br>-Experimental data.
SHIFTX<br><br>shiftx.wishartlab.com/	--	SHIFTX predicts ^{1}H, ^{13}C, and ^{15}N chemical shifts for proteins by using only its PDB file as input. On the web page, you can select if the protein is deuterated or not, the type of chemical shift to be predicted and the output file format.	+	Run from server.<br>Does not required installation.	-PDB.
SPARTA+<br><br>spin.niddk.nih.gov/bax/software	--	SPARTA+ employs a neural network algorithm to make chemical shift prediction from PDB files. To run Sparta+, you need the pdb file only, which must contain the coordinates of the hydrogen atoms or the decreases of prediction accuracy.	++	Download and decompress.<br><br>Command line program.	-PDB.
DichroCalc<br><br>comp.chem.nottingham.ac.uk/dichrocalc	--	DichroCalc is a web interface for protein circular and linear dichroism calculations. From a pdb file, you can generate different types of dichroism spectra by submitting the pdb file and selecting the type of file that will be generated and sent to an email address of your choice.	+	Run from server.<br>Does not required installation.	-PDB.

Software	Input data	Brief description	Difficulty to install and use[1]	Installation comments	Requirements
PDBMD2CD<br><br>pdbmd2cd.cryst.bbk.ac.uk/	--	PDBMD2CD predict circular dichroism spectra of proteins from a single PDB file or multiple models. Only the pdb files are needed.	+	Run from server.<br>Does not required installation.	-PDB.
PALES<br><br>spin.niddk.nih.gov/bax/software	--	Back-calculate residual dipolar couplings from a PDB file, it can also best-fit measured data to a structure.	++	Download and decompress.<br><br>Command line program.	-PDB.
ProtSA<br><br>webapps.bifi.es/protsa	--	Generate an assembly of unfolded proteins and calculating the average solvent-accessible surface area (SASA) of each atom and residue in the ensemble from the sequence of the protein. In addition, if you provide a PDB, folded SASA and differences between the folded and unfolded SASA, per atom and per residue, will be calculated.	+	Run from server.<br>Does not required installation.	-Amino Acid Sequence.<br><br>-PDB (optional)

1- Difficulty Scale:
+ very easy. ++ easy ; +++ moderate ; ++++ difficult
